# Genome access and other web-based IT solutions: Genetic counseling in the digital era

**DOI:** 10.3389/fpubh.2022.1035316

**Published:** 2022-11-07

**Authors:** Alessia Cazzaniga, Marta Plebani, Marco Crimi

**Affiliations:** Kaleidos SCS onlus, Scientific Office, Bergamo, Italy

**Keywords:** genetic counseling, genetic healthcare, telemedicine, digital health revolution, genomics services, genetic rare diseases

## Abstract

Genetic counselors are healthcare professionals who are trained in both medical genetics and counseling to help guide individuals through what is known about genetic predispositions toward a variety of diseases, how they are inherited, and what impact this information can have on them and their partners and families. The range and scope of practice of GC has greatly expanded beyond where it started and now, it is employed in a variety of clinical and research settings. The traditional approach to GC involves meeting with a counselor in person. However, with the increasing availability of online resources, more people are seeking information about genetic disorders online. This shift has led to the development of online GC services. Indeed, genetic counselors are no strangers to improvements in terms of adopting digital solutions in their clinical routine, however, there are few studies assessing genetic counselors' attitudes regarding existing digital tools. Genome Access^®^ is a digital platform that improves patient knowledge in the field of genetic diseases and supports specialists throughout different stages of counseling. This study aims to present Genome Access and discuss the importance of adopting digital technologies designed specifically for GC and what tools these solutions should include.

## Introduction

### Background and rationale

Clinical genomics is a fast-growing field and the simple access to the market of DNA testing (better known as “consumer genetics”) is becoming easier. In fact, we are facing a global-wide increased demand for genetic healthcare services that sometimes exceeds the clinical capacity (further exacerbated by the COVID-19 pandemic) (1). Therefore, there is a need for effective digital tools to improve the efficiency of remote delivery of “virtual” genetic care and to collect genomic data that can be analyzed through automated clinical interactions. To address this clinical need, this study aims to compare the effectiveness, usability, and acceptability of AI-based available platforms for delivering genetic care. Kaleidos, a no-profit Italian scientific research enterprise, has recently released “Genome Access,” a platform developed to “virtualize” several steps of the GC process to help people improve their knowledge on their genetic health status and become more confident to make decisions (2).

### Solution overview

Genome Access uses a variety of AI technologies to manage the GC process, including an automated conversation model (chatbot) to enable contextual conversations on genetics. Together, these technologies cover the input, processing and analysis, and the output needed during GC. Genome Access is available as a SaaS (Software as a Service) stand-alone system.

## Subsections relevant for the subject

### Benefits of the genome access solution

Kaleidos has recently released a digital solution fully compliant with privacy regulation (GDPR) and embedded with protocols entirely encrypted to protect users' personal identity. Genetic services facilities represent a perfect launching point for further development of digital solutions, as they characterize many routine telemedicine care processes that could be similarly automated. Moreover, Genome Access is expected to bring significant benefits for geneticists and healthcare staff by reducing their clinical burden. The Platform also has several benefits for patients. Genome Access will provide a reliable and consistent safety net before in-person visits (when needed). Patients will be able to ask questions about their risk recurrency just as they would with a in person clinician. The system is convenient because it does not require them to travel to the site of care and can be used at the time and/or for the duration that suits them.

### Genome access's key features

**Figure 1:**
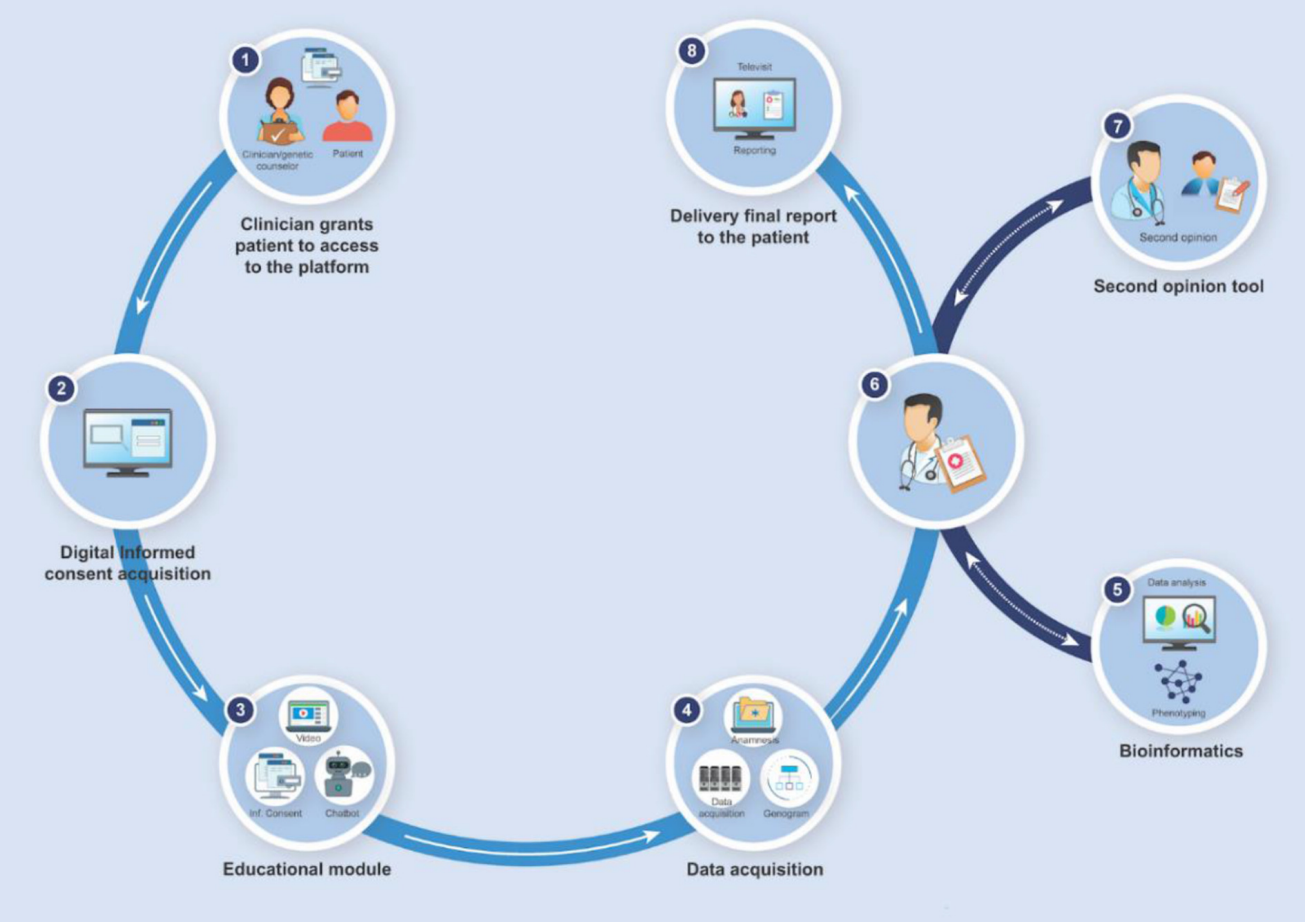
Workflow map of the digitalized steps on a GC service. (1) The access to the private area is allowed only by one-time password (OTP); (2) The platform manages the dematerialized Informed Consent through eSignature; (3) The “Training” module focuses on the basis of genetics: patients can query “Gene-bot,” a virtual assistant *ad-hoc* built for answering on diseases due to DNA mutations; (4) Through a simple and guided procedure, the user provides personal and familiar data (including also the pedigree) which will be then acquired by the specialist; (5) Genome Access provides the specialist with advanced bioinformatics applications for data analysis and phenotyping; (6) The platform guides the specialist on the prescription step, with the possibility of (7) engaging a colleague for a specific second opinion consultation; (8) Thanks to the “Televisit” module, integrated within the platform, it is possible to assist patients remotely and optimize the clinical reporting.

The platform is based on innovative AI algorithms, specifically developed to revolutionize the counseling process for patients affected by genetic diseases (Figure 1). Genome Access is composed of three interoperable modules:

1. Information and Education. Genome Access is committed to finding innovative and more efficient ways to counsel and educate patients. Therefore, the goal behind the first module aims to streamline, by digitalization, the process of delivering privacy information, collecting the patient's consent and letting them have a better understanding of DNA tests results and to understand what they mean. Indeed, patients can consult “Genebot,” a custom chatbot made for genetics, developed to provide a platform for individuals to ask questions relevant to genetic testing. This includes understanding the meaning of a genetic test, or what to expect from genetic testing, as well as information about genetic disorders or conditions. Patients can also enjoy several interactive videos realized for genetics and DNA pathogenic variants risk recurrence.
2. Data retrieval. The second module provides tools for guided self-anamnesis and for collecting the pedigree. Moreover, the second module provides digital support to the professional in the genetic “raw” data analysis and phenotyping through bioinformatic software and specific algorithms.
3. Tele-genetics. This is an interoperable telemedicine solution aimed at virtualizing the relationship between patients and healthcare providers through a televisit system as well as offering software for elaborating a final clinical report. Genome Access can easily be implemented by a variety of customers, such as clinical facilities, patient advocacy organizations of rare diseases, laboratories for molecular analyses, and research centers: the platform can be personalized to meet the specific needs of our customers for developing custom-tailored solutions. Moreover, Genome Access can be easily integrated into third-party applications through API applications.

#### Technology in the genetic counseling setting

##### Medical history taking

With pioneering technological advancements, such as tools for drawing electronic pedigrees (genogram), geneticists have digitalized the process of collecting relevant personal, family, and clinical information, reducing the time spent on these duties during in-person appointments. The digitalization of the medical history taking procedure resulted in genetic counselors being able to more easily retrieve complex family histories, to review relevant information more quickly, and to reduce appointment times for patients (3).

##### Digital questionnaires

Many clinics send questionnaires to patients before their appointments; in most cases these forms are even accessible online so that patients do not have to wait for a mailed copy (4).

##### Telegenetics software

There is an increasing number of private healthcare companies that are employing counselors specifically to provide telemedicine (5). Televisit enables genetic professionals to counsel patients at home. Some genetics clinics have adopted digital tools to deliver pre-test information, followed by shorter sessions to discuss other recommendations or testing options tailored toward patients' specific personal and/or family histories (6).

##### Chatbots

A chatbot is a computer program that uses AI and natural language processing to simulate conversations with “human” users. There are currently several chatbots that have been developed and are in use by genetic counselors. Chatbots are already being used as part of patient-initiated testing journeys, to allow patients to give consent to participate in research studies, and to provide valid support to counselors on disclosing positive genetic test results to patients (7) and discussing with them therapeutic options. Nevertheless, chatbots should not be intended as a delivery method alone for the return of genetic tests. Chatbots can be also used to support patients in scheduling an appointment with a genetic counselor. Usually, the chatbot delivers a feedback to users through pre-written answers (provided by a multidisciplinary team of genetic counselors); in the event that there is a question that does not have a pre-programmed response, the query is forwarded to the care team, who then creates an answer for the chatbot to be included in future interactions.

#### Comparison between available digital platforms of genetic counseling

Besides Genome Access, several genomic platforms have flourished around the world in the past few years with the same goal: to streamline genetic counseling through the digitization of time-consuming processes and, in most cases, with the support of AI tools. Digital solutions in genetic counseling fall into two main categories: platforms that offer both direct and remote counseling digitally delivered by “in-person” counselors and web-based software offering a variety of services, such as ancestry tracing, genetic health risk assessment, and carrier screening. There are already available some different digital platforms focused on genetic counseling: some clear differences between them are highlighted (Table 1). Most of those platforms are based on searchable databases of genetic conditions, which can be helpful for those who are seeking information about their health or the health of their relatives. In some cases, the database also provides information on drug interactions and how different genes interplay together. For most of the platforms, the users can create their accounts to store their information, and, in addition, some platforms have social networking components, which allow users to connect with others who have similar conditions. This tool could be very helpful to improve networking especially in the rare diseases field (8). It can be difficult to decide which platform is the right for the user's needs. To make an informed decision, it is important to compare the different services offered by each platform, especially regarding the health risk assessments.

**Table 1:** Digital genetic platforms comparison.

**Digital** ** solution**	**Genome** ** Access**	**Igentify**	**FDNA (Face2Gene)**	**OptraHealth (GENEFax)**	**GeneMatters**	**Invitae**	**MediFIRST**	**Phenotips**	**My Gene Counsel**	**TellmeGen**
**Main focus**	IT solution for medical genetics	IT solution for medical genetics	Detection of phenotypes from facial photos	IT solution for medical genetics	To deliver telehealth genetic counseling	IT solution for medical genetics	IT solution for medical genetics	IT solution for medical genetics	To deliver telehealth genetic counseling	Direct-to-consumer DNA testing
**Model of** **service**	B2B2C	B2B	B2C	B2B	B2B & B2C	B2C	B2B	B2B	B2B & B2C	B2C
**Headquarter**	Italy	Israel	U.S.A.	U.S.A.	U.S.A.	U.S.A.	France	Canada	U.S.A.	Spain
**Genetic area**	Genetic diseases in general	ECS, PGX, and NIPT	Mostly pediatrics	Genetic diseases in general	Oncology, reproductive health, pediatrics, cardiology, and neurology	Reproductive health, pediatrics, oncology, and cardiology	Reproductive health and genetic diseases in general	Pediatrics and oncology	Genetic diseases in general	Genetic diseases in general and pharmacogenetics
**Remote** **in-person** **meetings opt**.	Yes	No	No	Yes	Yes	Yes	No	No	No	Yes
**Direct** **DNA testing**	No	No	No	No	No	Yes	No	No	No	Yes
**Main** **available digital modules & tools**	- Chatbot - Education videos - Genogram and anamnesis - Phenotyping - Test prescription - Televisit - OTP login to private area	Genetic data analysis - Genogram - Education videos	- Next Generation Phenotyping technology (for people without diagnosis) - Networking component among multidiscip linary experts	Remote patient triaging - Genogram - Personalized risk assessment - Real-time recommen dations on tests - Chatbot - Digital return of results	Televisit - Education modules - Follow-up monitoring - Patient decision-making supporting tool	Chatbot - Carrier screening - Personalized medication management - Prenatal diagnostic testing - Testing support and education	Assisted Reproductive Technology - Genogram - Follow-up monitoring - Online prescriptions	Genogram - Phenotyping - Pre-test questionnaires - Cancer risk assessment	Chatbot - Genetic health risk assessment - Results delivery, with algorithm for DNA variants reclassification	Carrier screening - Ancestry tracing - Phenotypic traits
**Other relevant features**	- IPR - HL7/FHIR compliance - White-label solution	- Custom dynamic videos - Specific solutions for genetic labs	HIPAA compliance - Deep learning algorithms	Searchable database of genetic conditions and QAs - Custom options	SOC2 compliance - Multilingual counselor' availability - EMR-integrated software	HIPAA compliance - At home saliva collection	App for smartphones, available for e-prescriptions - Specific solutions for laboratories	- HIPAA compliance - EMR-integrated software	- Several trademarks obtained	FDA revised - GCP, cGMP, GLP, ISO compliance/cert ifications - At home saliva collection

Comparison of some of the best-known digital solutions currently available. Disclaimer: the information mentioned for the comparison listed above was collected exclusively using publicly available and easily retrievable resources at the time of publication on each company website.

B2B, business-to-business; B2C, business-to-consumer; ECS, expanded carrier screening; PGX, pharmacogenomics; NIPS, non-invasive prenatal screening.

#### The protection of genetic data in the digital era

The pattern of informed consent acquisition on the use of genetic data is becoming more and more prevalent as technology advances. However, while the benefits of using such data are clear, the privacy measures that need to be applied are not always given the attention they deserve (9). Genetic data can reveal a lot of information about an individual, including their health, ancestry, and predispositions to certain diseases. As such, any solution that digitally collects, analyzes, and stores DNA-related data must take steps to protect the privacy of the users. This includes implementing appropriate technical safeguards (based on privacy “by design” principles), as well as ensuring that individuals have a clear understanding of how their data will be used and what rights they have about it. If those concerns are not properly taken into account, these same advancements could lead to serious invasions of privacy.

## Discussion

Technological advances have already served to help genetic counselors to see more patients and spend less time on repetitive steps of face-to-face appointments and facilitate the access to genetic counseling services to patients (10). As in other fields, technology does not diminish the professionality of genetic counseling, since counselors have adopted it to serve both their needs and the needs of their patients (11). Digital genetic platforms have the potential to be very helpful for genetic counselors, increasing both efficiency and the ability to see more patients. However, it is crucial to always guarantee to genetic counselors the opportunity and ability to decide independently what digital tools (and uses) to be implemented in their services. Genome Access covers a wide range of topics, from prenatal to cancer to general genetics, and, similarly to other available digital genetic platforms, offers a number of advantages over traditional approaches. First, it provides people with access to information about genetic disorders 24 h a day, 7 days a week. Second, it allows people to communicate with health providers at their convenience. Third, it makes it possible for people to receive counseling regardless of where they are geographically located. Finally, it is easy to use. Despite these general advantages, there are some disadvantages to digital genetic counseling platforms. First, not all disorders are suitable for digital counseling. Second, not all counselors are qualified to provide online services. Third, not all information available online is accurate or up-to-date.

## Data availability statement

The original contributions presented in the study are included in the article, further inquiries can be directed to the corresponding author.

## Author contributions

AC and MP contributed both to draft the manuscript and performed data collection. MC conceived and designed the manuscript, supervised data collection, and drafted the manuscript. AC, MP, and MC confirm that they had full access to all the data in the study and take responsibility for the integrity of the data and the accuracy of the data analysis. All authors gave final approval of this version to be published and agree to be accountable for all aspects of the work in ensuring that questions related to the accuracy or integrity of any part of the work are appropriately investigated and resolved and approved the final manuscript.

## Funding

This study was supported by the grants Artemisia (CESVI-Bergamo) and SI4.0 2021 (Unioncamere Lombardia) awarded to MC.

## Conflict of interest

Authors AC, MP, and MC were employed by Kaleidos SCS onlus.

## Publisher's note

All claims expressed in this article are solely those of the authors and do not necessarily represent those of their affiliated organizations, or those of the publisher, the editors and the reviewers. Any product that may be evaluated in this article, or claim that may be made by its manufacturer, is not guaranteed or endorsed by the publisher.

## Abbreviations

AI: artificial intelligence
GC: genetic counseling
GDPR: general data protection regulation
API: Application Programming Interface.
